# Prevention of Infections in Cardiac Surgery study (PICS): study protocol for a pragmatic cluster-randomized factorial crossover pilot trial

**DOI:** 10.1186/s13063-018-3080-y

**Published:** 2018-12-17

**Authors:** Rachel B. van Oostveen, Alberto Romero-Palacios, Richard Whitlock, Shun Fu Lee, Stuart Connolly, Alex Carignan, C. David Mazer, Mark Loeb, Dominik Mertz

**Affiliations:** 1 Population Health Research Institute (PHRI), Hamilton Health Sciences, Hamilton, ON Canada; 2 McMaster University, Hamilton, ON Canada; 3 Department of Microbiology and Infectious Diseases, Université de Sherbrooke, Sherbrooke, QC Canada; 4 Centre de recherche du Centre hospitalier universitaire de Sherbrooke, Sherbrooke, QC Canada; 5 Li Ka Shing Knowledge Institute of St. Michael’s Hospital, University of Toronto, Toronto, ON Canada; 6 Juravinski Hospital and Cancer Center, 711 Concession Street, Section M, Level 1, Room 3, Hamilton, ON L8V 1C3 Canada

**Keywords:** antibiotic prophylaxis, cardiac surgery, cluster crossover trial

## Abstract

**Background:**

A wide range of prophylactic antibiotic regimens are used for patients undergoing open-heart cardiac surgery. This reflects clinical equipoise in choice and duration of antibiotic agents. Although individual-level randomized control trials (RCT) are considered the gold standard when evaluating the efficacy of an intervention, this approach is highly resource intensive and a cluster RCT can be more appropriate for testing clinical effectiveness in a real-world setting.

**Methods/design:**

We are conducting a factorial cluster-randomized crossover pilot trial in cardiac surgery patients to evaluate the feasibility of this design for a definite trial to evaluate the optimal duration and choice of perioperative antibiotic prophylaxis. Specifically, we will evaluate: (a) the non-inferiority of a single preoperative dose compared to prolonged prophylaxis and (b) the potential superiority of adding vancomycin to routine cefazolin in terms of preventing deep and organ/space sternal surgical site infections (s-SSIs). There are four strategies: (i) short-term cefazolin, (ii) long-term cefazolin, (iii) short-term cefazolin + vancomycin, and (iv) long-term cefazolin + vancomycin. These strategies are delivered in a different order in each health-care center participating in the trial. The centers are randomized to an order, and the current strategy becomes the standard operating procedure in that center during the study.

The three feasibility outcomes include: (1) the proportion of patients receiving preoperative, intra-operative, and postoperative antibiotics according to the study protocol, (2) the proportion of completed follow-up assessments, and (3) a full and final assessment of the incidence of s-SSIs by the outcome adjudication committee.

**Discussion:**

We believe that a cluster-randomized factorial crossover trial is an effective and feasible design for these research questions, allowing an evaluation of the clinical effectiveness in a real-world setting. A waiver of individual informed consent was considered appropriate by the research ethics boards in each participating site in Canada as long as an information letter with an opt-out option was provided. However, a waiver of consent was not approved at two sites in Germany and Switzerland, respectively.

**Trial registration:**

Clinicaltrials.gov, NCT02285140. Registered on 15 October 2015.

**Electronic supplementary material:**

The online version of this article (10.1186/s13063-018-3080-y) contains supplementary material, which is available to authorized users.

## Background

In 2016, 295,705 cardiac surgical procedures were performed in the U.S. [1]. Despite routine antibiotic prophylaxis use in cardiac surgery, the incidence of sternal surgical site infections (s-SSI) ranges from 1% to 10% [2–5]. S-SSIs. Deep and organ/space s-SSIs contribute significantly to the complications that can follow surgery, resulting in a prolonged duration of hospital stay, higher mortality rates, increased health-care costs, and decreased patient satisfaction [2].

### Clinical equipoise in duration of antibiotic prophylaxis in cardiac surgery

It is clear that antibiotic prophylaxis is beneficial for preventing s-SSIs in cardiac surgery. However, there is no consensus on the optimal duration of prophylactic administration or the antibiotic to be used, resulting in a wide range of practices and recommendations in clinical practice guidelines [2, 6–10], where the recommendations vary from a single dose [7] to doses being repeated for up to 72 h [6]. The lack of agreement in clinical practice guidelines is reflected in a variety of approaches being used at the local level, which was demonstrated in a survey conducted by our group at 11 Canadian cardiac surgery centers in 2011 (unpublished data). In this survey, six centers reported continuing antibiotic prophylaxis for up to 24 h, and five centers continuing beyond 24 h with one center providing coverage for greater than 48 h. This wide variety in the duration of prophylactic regimens used in clinical practice and recommended in clinical practice guideline points towards a lack of convincing evidence supporting any approach. Although systematic reviews found that antibiotic prophylaxis for ≥24 h may be more beneficial in preventing s-SSIs [11, 12], no definite conclusions can be drawn due to the risk of bias and the heterogeneity in antibiotics used [11]. This highlights the need for a definite, sufficiently powered and rigorous randomized controlled trial (RCT) to provide the evidence that is currently lacking.

### Clinical equipoise in choice of antibiotic prophylaxis in cardiac surgery

In conjunction with the lack of consensus on the duration of prophylaxis, the antibiotic used varies across cardiac centers, and an increasing number of centers are routinely using glycopeptides in addition to routine cephalosporins [13–15]. The use of cephalosporins as the first-line antibiotic for prophylaxis in surgery, as recommended in recent guidelines [5, 7], is supported by systematic reviews and meta-analyses that demonstrate that beta-lactams are superior to glycopeptides alone in preventing s-SSIs after cardiac surgery [12, 16, 17]. On the other hand, glycopeptides have been shown to prevent infections by pathogens resistant to routine cephalosporin prophylaxis by reducing the incidence rates of s-SSIs due to methicillin-resistant *Staphylococcus aureus* (MRSA) [18] and coagulase-negative staphylococci [19]. Furthermore, a recently published cohort study showed a reduction in the number of cases of SSIs with a combination treatment of a beta-lactam plus vancomycin in cardiac surgery patients [15]. While the superiority of cephalosporins does support the routine use of this group of antibiotics, that a combination with glycopeptides reduced SSI rates (in particular, due to cefazolin-resistant pathogens) suggests the use of a combination of both groups of antibiotics. Along these lines, a recent meta-analysis called for high-quality trials to assess further the benefit of vancomycin as an additional component of the prophylactic bundle to prevent s-SSIs [20].

This article describes the rationale, design consideration, and experience to date with the Prevention of Infections in Cardiac Surgery (PICS) trial, which is evaluating the effect of duration and choice of antibiotic on the incidence of s-SSIs. This study is currently in a pilot phase to assess the feasibility of such a trial.

## Methods/design

### Study design

In this factorial cluster crossover pilot study, there are four strategies for prophylactic antibiotic use: (i) short-term cefazolin (A), (ii) long-term cefazolin (B), (iii) short-term cefazolin + vancomycin (C), and (iv) long-term cefazolin + vancomycin (D; Table 1). Each participating hospital will use each of the four strategies in a specific order. We have defined eight possible orders: ADBC, ADCB, DABC, DACB, BCAD, BCDA, CBAD, and CBDA. The study site is randomized to one of the eight orders with a probability of 1/8. The randomization sequence was generated using SAS V9 and is kept by a researcher not involved in the trial.

**Table 1:** Antibiotic regimens used in the four study arms

	Short-term arm	Long-term arm
Mono-prophylaxis	Cefazolin 2–3 g^a^ preop Cefazolin 2 g at wound closure/4 h after the preop dose No post-op antibiotics	Cefazolin 2–3 g^a^ preop Cefazolin 2 g at wound closure/4 h after the preop dose Cefazolin 2 g q8h × 5 doses
Combination prophylaxis	Cefazolin as above Vancomycin 1–1.5 g^b^ preop No post-op antibiotics	Cefazolin as above Vancomycin 1–1.5 g^b^ preop, repeated q12h × 3 doses

Doses of both cefazolin and vancomycin will be adjusted for patients with known renal impairment

*Preop* preoperative, *q8h* every 8 h, *q12h* every 12 h

^a^Cefazolin 3 g if patient weight greater than 120 kg

^b^Vancomycin 1.5 g if patient weight greater than 85 kg

The initial strategy becomes the standard of practice for antibiotic prophylaxis in the participating hospital for 6 months. The subsequent strategies are revealed to the primary investigator and local study personnel only 1 month prior to the phase-in of the strategy, or as soon as required by the site to allow a smooth transition. These subsequent strategies become the standard of practice in that site for the following 6 months. The 1-month phase-in/wash-out period between strategies allows for effective implementation of the subsequent strategy and reduces potential contamination. Hence, the duration of the trial at each site consists of 6 months for each of the four strategies plus the 1-month phase-in period. The trial will be followed by a 3-month period to collect data, giving a total of 31 months.

**Fig. 1:**
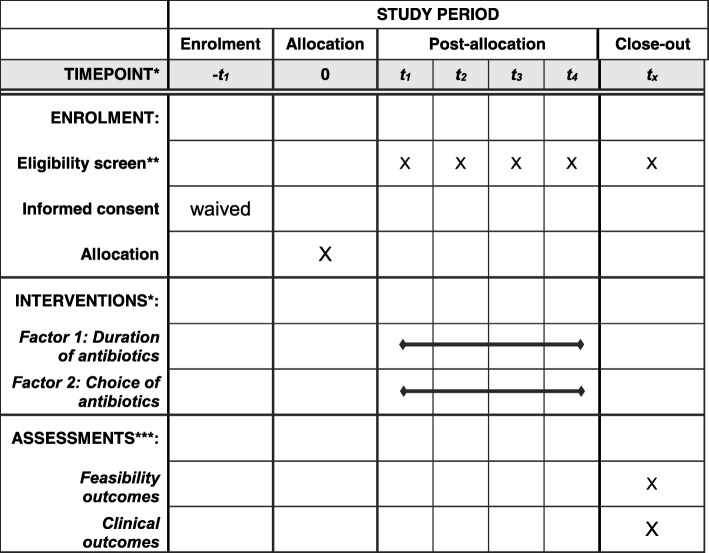
SPIRIT figure. *t1-t4: four study arms, order of the study arms for each study site allocated at timepoint ‘0’. **Exclusion criteria applied upon data collection. ***Data collection at least 90 days after surgery

For the pilot study, we recruited three hospital sites (Hamilton Health Sciences, Hamilton, ON, Canada; Sherbrooke, QC, Canada; and St. Michaels Hospital, Toronto, ON, Canada). If no major changes to the study protocol are needed, we are planning to include the data from the pilot study as part of the eventual full-scale trial (Additional file 1: SPIRIT checklist and Fig. 1).

The steering committee provided support and advice in terms of the study design and the clinically important minimal difference for the sample size calculation, and it will provide advice in terms of the next steps once the pilot study is completed.

### Eligibility criteria for centers and patients

Centers with a volume of >300 open-heart cardiac surgery procedures per year are eligible. All adult patients (≥18 years of age) undergoing open-heart surgery requiring midline sternotomy will be included in the study. Exclusion criteria are limited to: (1) use of therapeutic antibiotics at the time of surgery, (2) previous enrolment in this trial, (3) a beta-lactam or vancomycin allergy, (4) known MRSA carriage, or (5) participation in another study that may interfere with this trial. Given that a waiver of consent can be justified for this study (see ethical considerations below), no consent procedures are needed. No data will be collected on patients who opt out of the study.

### Hypothesis

The eventual full-scale trial will assess the efficacy of a combination of cefazolin and vancomycin compared to cefazolin monotherapy, and also compare long-term versus short-term prophylaxis with these regimens. We hypothesize that a combination of vancomycin with cefazolin is superior to cefazolin monotherapy and that short-term prophylaxis is as effective as long-term prophylaxis. If the short-term combination regimen is proven superior, this approach would reduce overall exposure to antibiotics while reducing infection rates.

### Outcomes

The pilot study was designed to assess the feasibility of the eventual full-scale trial. The pilot study is estimated to have 500 participants per strategy per site. Therefore, the three feasibility outcomes were:

1. The proportion of patients receiving antibiotics according to the study protocol as ascertained by a chart review of a random sample of 25% of the participants per site per strategy with a goal of greater than 90% with a 95% confidence interval (CI) of 85% to 95% based on an estimated sample size of 125 participants per site per strategy.
2. The proportion of patients who completed the follow-up at 90 days with a goal of ≥95% (i.e., less than 5% lost to follow-up) with a 95% CI of 93% to 97% based on an estimated sample size of 500 participants per site per strategy.
3. The assessment of the primary outcome of the eventual full trial (i.e., incidences of deep and organ/space s-SSIs) by the blinded outcome adjudication committee with an inter-rater kappa greater than 0.8.

If these criteria are met, then the eventual full-scale trial will be considered feasible.

For the eventual full-scale study, the primary outcome will be the composite of deep incisional and organ/space s-SSIs following Center for Disease Control and Prevention and National Healthcare Safety Network definitions [21], which are based on clinical and microbiological criteria of infection at the surgical site. These must appear to be related to the operative procedure and occur within 90 days following surgery. Secondary outcomes include all types of s-SSI (including superficial incisional infections) at the 90-day follow-up as well as laboratory-confirmed *Clostridium difficile* infections and mortality in patients with an active infection. Data on these clinical outcomes are collected during the pilot phase of this study as well.

### Data collection

Assessment of study outcomes is primarily based on routinely collected data. Patients are seen every day by their attending physician during their hospital stay. Furthermore, patients are typically seen approximately 1 month after surgery by their surgeon to address any ongoing or new issues. Finally, participants will be contacted by phone by research staff at least 90 days after surgery unless a routine clinic visit occurred after the 90-day mark. If a patient develops any signs or symptoms of a sternal infection, a case report form will be created, documenting the course and treatment of the infection. These case reports will then be reviewed by a blinded outcome adjudication committee to determine whether a s-SSI occurred.

We are using RedCap, a secure online research database, for data collection. The keys used to link study numbers with the patients are kept at the local study sites on secure password-protected computers. After completion of the trial, only the principal investigator will have access to the data, and they may grant access to others if required.

### Sample size calculations

Three study centers with a total of roughly 6000 patients combined will be enrolled for the pilot study. To reflect the clustered and crossover nature of the design, the 95% CIs are constructed to ensure that each strategy at each site reaches the feasibility goals, rather than having an overall level across all sites and strategies. While such a large sample size for feasibility outcomes is not necessarily needed, we must enroll this number of participants in at least two or three sites to gain the necessary insight into potential challenges with the feasibility of the study design for the full trial.

For the eventual full trial, we assume a true deep and organ/space s-SSI rate of 2.0%, and a loss to follow-up and death of 5% each. A total of 16 hospital sites with an average of 1000 patients undergoing cardiac surgery per year would need to be enrolled to give 80% power to detect a reduction in s-SSIs of 0.6% (±30% relative reduction) at a significance level of 5% (two-sided). The same sample size would provide 95% power to detect an increase of s-SSIs with the short-term prophylaxis strategy of 0.55% (±27.5% relative increase, 5% significance level, one-sided). The sample size was calculated using the formula derived by Giraudeau et al. [22] and first expressed in Connolly et al. [23]. We assume that the interperiod correlation (IPC) is 0.9 times the intraclass correlation (ICC). We chose a conservative estimate of the ICC of 0.0018 based on the observed value of ICC in the PADIT trial [23], which results in a larger sample size for the range of ICCs and IPCs tested.

### Statistical analysis

Feasibility outcomes for the pilot study will be reported descriptively. For clinical outcomes, i.e., the outcome of the eventual full-scale trial, we will apply hierarchical modelling (a generalized linear mixed model) for binary outcomes adjusted for the cluster-period effect, as proposed by Turner et al. [24], and stratified according to the factorial allocation. The association will be reported by the means of odds ratios and a 95% CI. If no major changes to the study protocol are needed, we will include the pilot study as part of the eventual full-scale trial. The study will be analyzed using an intention-to-treat approach and patients lost to follow-up will be excluded.

## Discussion

### Rationale for a factorial cluster crossover randomized trial

Individual-level RCTs are considered to be the gold standard in the evaluation of interventions [25] and are needed for regulatory purposes to show the efficacy of a drug under highly controlled conditions in a highly selected group of patients. Therefore, findings from these RCTs do not necessarily translate into effectiveness in a real-world setting. In particular, individual-level RCTs are not well suited for studying system changes, and a cluster design may be more appropriate as this design aligns research with clinical care, allowing enrolment of a representative patient population in a real-world setting [23, 26, 27]. Cardiac surgery is conducted in specialized centers using highly standardized procedures, an approach that lends itself to a cluster RCT design. In our study, the antibiotic prophylaxis regimen to be studied becomes part of the standardized operating procedures for a certain period for all patients undergoing cardiac surgery at the participating centers.

In addition to the study goal to evaluate effectiveness in a real-world environment rather than efficacy, a trial randomizing individual patients would likely not be feasible due to financial constraints, considering the large sample size needed to power the study properly, in the absence of any interest from industry to support such a trial. In contrast, if the randomization occurs at the level of the health-care center, and therefore, the study intervention becomes the standard operating procedure for that center, the resources required are significantly reduced.

Similarly, we chose to incorporate a factorial component to evaluate two different interventions (choice and duration of antibiotics, respectively) without requiring a substantial increase in sample size. This is an important advantage of a factorial design, especially when resources are limited [28]. Furthermore, it has been suggested that using a factorial design is particularly beneficial when the purpose of the study involves choosing an optimal intervention [29].

Finally, the crossover element of the study allows each center to act as its own control. This approach is proposed to protect from trends over time that are unrelated to antibiotic prophylaxis, such as possible shifts in the epidemiology of s-SSIs, while avoiding a negative impact on the power of the study.

### Risk of bias

The antibiotic prophylaxis strategy at any given time will be known to the health-care providers, as the strategy will be the standard procedure for all patients undergoing cardiac surgery at each participating center. Thus, patients as well as health-care staff cannot be blinded to the antibiotic prophylaxis strategy. This is the most important potential source for bias using this study design (detection and ascertainment bias). To protect against this, a blinded outcome adjudication committee (consisting of experts not involved in the care of the study participants) will determine the presence of a s-SSI for each participant based on case reports prepared by the research staff. Furthermore, a universally accepted standard definition of surgical site infection will be used [21]. To avoid subjectivity that may put outcome ascertainment at risk of bias, the definition will be adapted such that diagnosing deep incisional/organ/space s-SSIs is not done by the surgeon or attending physicians. Similarly, superficial incision infections will not be considered a primary outcome due to the subjectivity in assessing this outcome as well as the lack of clinical importance in comparison to deep and organ/space s-SSIs.

### Ethical considerations

Obtaining individual consent for this study would jeopardize one of the main advantages of the study design, that is, testing the comparative effectiveness of the interventions in a real-world setting rather than testing efficacy in a subgroup of highly selected patients. Similarly, obtaining individual patient consent would not allow assessment of the intervention in important groups of patients, such as emergent and urgent surgery patients or those from diverse ethnic backgrounds who may not be proficient in the official languages of Canada.

The Canadian Tri-Council Policy Statement allows research ethics boards to approve an alteration to the informed consent process, such as a waiver of consent, if the following criteria are met: (1) there is no more than minimal risk to participants, (2) the alteration to consent requirements is unlikely to affect the welfare of participants adversely, (3) it is impossible or impracticable to carry out the research properly given the research design if prior consent is needed, and (4) there is a plan to offer participants the possibility of having their data deleted from the study database [30]. Similar criteria are used in the United States [31].

Considering that both antibiotics as well as the durations used in this study are considered the standard of care in some centers, we strongly believe that our trial meets the first criterion required for a waiver of individual informed consent as the risk to participants is perceived to be minimal. The duration and antibiotics chosen in this clinical trial represent either current practice at some centers or are in keeping with current guidelines. The variety of antibiotic practices across Canada and elsewhere supports the clinical equipoise surrounding antibiotic choice and duration in cardiac surgery. Thus, the risk for participants will be minimal and the risk related to the surgical procedure is identical in all groups. Cefazolin and vancomycin are routinely used and have a favorable safety profile when used for a short of time (see Discussion section on Safety).

A maximum of 48 h of antibiotic prophylaxis was chosen for the long-term strategies as previous studies have shown the risk of developing an infection with resistant pathogens increases with antibiotic use longer than 48 h [32]. Similarly, the extended duration of the use of antibiotics potentially increases the risk of other adverse events, such as diarrhea or infection with *Clostridium difficile.* However, there is no clear evidence that the risk is significantly higher with a 48-h course versus a shorter course of antibiotic prophylaxis. This possible adverse effect highlights the need to conclusively compare short- and long-term antibiotic prophylaxis.

The second requirement to waive consent is that this alteration is unlikely to affect the welfare of the participants adversely. This criterion is met in the study as the antibiotic regimens being investigated are in use across Canada or follow current guidelines. The choice and duration of antibiotics are not decisions made by patients, but by surgeons or anesthesiologists, usually by following internal guidelines and policies. As such, participation in the trial does not negatively affect the patient’s autonomy. If patients have a strong opinion about the antibiotic regimen they would like to receive, the clinician is free to overrule the recommended study regimen.

Thirdly, obtaining consent from individual participants for the antibiotic prophylaxis strategy would not be feasible in a cluster randomized controlled trial, as the protocol would need to be applied to all patients undergoing cardiac surgery during the study period. The resources required to obtain individual consent would be quite significant, including increased study personnel for recruitment and resulting costs. Furthermore, obtaining consent can be difficult in emergent and urgent procedures. Such patients are often excluded from data collection and analyses, which could affect the extrapolation of study results to this population of participants. For these reasons, it would be impracticable to carry out the research properly given the study design if prior informed consent was required from every participant.

Lastly, to meet the final criteria to waive informed consent, patients will be informed of the study while still in hospital before their data has been collected by study personnel, and again during the telephone follow-up call at 3 months after surgery. The patients are provided with contact details for the study coordinator, whom they can contact any time with questions around the study as well as to opt out from data collection.

Therefore, a waiver of consent or an opt-out option for patients is considered appropriate in this study of the clinical effectiveness of the current standard of practice for antibiotic regimens [33]. The research ethics boards at the pilot sites have agreed that these criteria are met and have waived the need for individual patient consent. However, the board at the first site insisted on an information letter with an option to opt out of data collection, which was also found acceptable by the other two research ethics boards. However, two ethics boards in Europe (Zurich, Switzerland, and Bavaria, Germany) concluded that the criteria for a waiver of consent are not met and would have insisted on essentially applying the same rules to this clinical effectiveness trial as for a phase III clinical trial.

In summary, the acceptable consent model for a clinical effectiveness trial using a cluster RCT design may differ from one jurisdiction to the next, and researchers must be aware that the approval given by one research ethics board will not necessarily translate into approval in other jurisdictions. Given the research question and study design, we aim to recruit only study sites that are able to obtain a waiver of consent for the reasons summarized above.

In accordance with good clinical practice and expectations by research ethics boards, all protocol modifications are communicated to investigators and ethics boards, and the registration on clinicaltrials.gov is updated.

### Safety

Vancomycin has well-known adverse effects of nephrotoxicity and ototoxicity; however, these adverse effects have virtually never been reported with trough levels lower than 15 mg/L and 50 mg/L, respectively. Trough levels after one single dose will remain below this toxic level; thus, a single dose is very unlikely to cause significant renal injury or ototoxicity. Even when three more doses are administered in the long-term strategy, these side effects are highly unlikely and a steady state would not yet have been reached. However, due to concerns by some surgeons, perioperative physicians, and intensivists, vancomycin levels have been occasionally been measured in patients that they deemed at risk of kidney injury. In the light of the availability of these data and a recent study that found an increase in acute kidney injury with combination prophylaxis [15], we will conduct a review of postoperative vancomycin levels that had been measured by intensivists during our longer-term combination treatment strategy at one of our study sites.

Given that only antibiotics that are routinely used for perioperative prophylaxis are administered as part of this study, a data safety and monitoring committee was not deemed to be necessary for the pilot study.

### Trial status

The current protocol version is 1.1. To date, we have completed all four strategies at two sites, with the third and final study site currently enrolling into the second strategy. There is currently data on 3986 patients in the database. The pilot phase will be completed once the third site completes enrolment on 28 February 2020.

## Additional file

Additional file 1: Spirit Checklist. (DOC 121 kb)

## Abbreviations

CI: Confidence interval
ICC: Intraclass correlation
IPC: Interperiod correlation
MRSA: Methicillin-resistant *Staphylococcus aureus*
PICS: Prevention of Infections in Cardiac Surgery
RCT: Randomized controlled trial
s-SSI: Sternal surgical site infection
