# Correction to: Prevention of Infections in Cardiac Surgery study (PICS): study protocol for a pragmatic cluster-randomized factorial crossover pilot trial

**DOI:** 10.1186/s13063-019-3771-z

**Published:** 2019-10-16

**Authors:** Rachel B. van Oostveen, Alberto Romero-Palacios, Richard Whitlock, Shun Fu Lee, Stuart Connolly, Alex Carignan, C. David Mazer, Mark Loeb, Dominik Mertz

**Affiliations:** 1 Population Health Research Institute (PHRI), Hamilton Health Sciences, Hamilton, ON Canada; 2 McMaster University, Hamilton, ON Canada; 3 Department of Microbiology and Infectious Diseases, Université de Sherbrooke, Sherbrooke, QC Canada; 4 Centre de recherche du Centre hospitalier universitaire de Sherbrooke, Sherbrooke, QC Canada; 5 Li Ka Shing Knowledge Institute of St. Michael’s Hospital, University of Toronto, Toronto, ON Canada; 6 Juravinski Hospital and Cancer Center, 711 Concession Street, Section M, Level 1, Room 3, Hamilton, ON L8V 1C3 Canada

**Correction to: Trials (2018) 19:688**

**https://doi.org/10.1186/s13063-018-3080-y**

Following publication of the original article [1], we have been notified of a few mistakes in the “Sample size calculations” section, second paragraph.

The phrase:

- “The same sample size would provide **95%** power to detect an increase of s-SSIs with the short-term prophylaxis strategy of **0.55% (±27.5%** relative increase, 5% significance level, one-sided).”

Should actually be:

- “The same sample size would provide **83%** power to detect an increase of s-SSIs with the short-term prophylaxis strategy of **0.8% (±40%** relative increase, 5% significance level, one-sided).”
